# NADPH Oxidase and Angiogenesis Following Endothelin-1 Induced Stroke in Rats: Role for Nox2 in Brain Repair

**DOI:** 10.3390/brainsci3010294

**Published:** 2013-03-19

**Authors:** Caroline J. Taylor, Robert M. Weston, Gregory J. Dusting, Carli L. Roulston

**Affiliations:** 1 Stroke Injury and Repair Team, O’Brien Institute, 42 Fitzroy St, Fitzroy, Melbourne, Victoria 3065, Australia; E-Mails: cj.taylor@unimelb.edu.au (C.T.); robmweston@gmail.com (R.M.W.); 2 Cytoprotection Pharmacology Program, Centre for Eye Research, The Royal Eye and Ear Hospital Peter Howson Wing, Level 1, 32 Gisborne Street, East Melbourne, Victoria 3002, Australia; E-Mail: g.dusting@unimelb.edu.au; 3 Department of Opthamology, Faculty of Medicine, University of Melbourne, Melbourne, Victoria 3010, Australia; 4 Department of Surgery, Faculty of Medicine, University of Melbourne, Melbourne, Victoria 3010, Australia

**Keywords:** cerebral vascular regeneration, free radicals, brain repair, endothelin-1 model

## Abstract

NADPH oxidases contribute to brain injury, yet they may also have a role in brain repair, particularly in vascular signaling and angiogenesis. This study determined the temporal and spatial profile of NADPH oxidase subunit expression/activity concurrently with angiogenesis in the brain following transient ischemic stroke induced by prolonged constriction of the middle cerebral artery by perivascular injection of endothelin-1 in conscious Hooded Wistar rats (*n* = 47). VEGF mRNA expression was increased in the ipsilateral cortex and striatum between 6 h and 28 days post-stroke concurrently with a marked increase in Nox2 mRNA expression up to 7 days, and increased Nox4 mRNA expression detected between 7 and 28 days. Point counting of blood vessels using Metamorph imaging software showed increased vascular sprouting between 3 and 7 days after stroke with new vascular networks detected in the core infarct region by 14 days. Angiogenic blood vessels 3 and 7 days post-stroke were observed to co-localise with both Nox2 antibody and dihydroethidium fluorescence suggesting a role for Nox2 generated superoxide during the phase of vascular remodeling, whilst Nox4 expression was detected once new cerebral vessels had formed. These results indicate for the first time that ROS signaling through a cerebrovascular Nox2 NADPH oxidase may be important in initiating brain angiogenesis.

## 1. Introduction

A slow but consistent recovery can be achieved in adult stroke patients during rehabilitation over months to years without pharmaceutical intervention [1]. The injured brain is primed for repair processes and ischemic insults to the brain have now been shown to trigger progenitor cell proliferation and stem cell migration from the sub ventricular zone (SVZ) of the lateral ventricle to damaged regions of the brain [2]. More importantly, stroke-induced neurogenesis occurs in the adult human brain, even in patients of advanced age [3]. For these reasons, neurorestorative strategies to facilitate functional recovery represents a new and promising area for stroke treatment. Fundamental to their development is the need to establish a supportive microenvironment in the brain after stroke that can support brain plasticity and neurorepair [4].

Angiogenesis is a fundamental process occurring both during development and in wound healing in adults. Migration of neural progenitor cells from the SVZ is tightly coupled with angiogenesis [5,6] and there is a correlation between angiogenesis and improved functional outcome after stroke even in human patients [7,8]. Signals generated from endothelial cells within compromised vessels adjacent to the ischaemic region serve as stimuli promoting stem cell migration, which in turn produce angiogenic factors, creating a loop of vascular stem cell interactions [6]. Injured blood vessels, activated endothelial cells, and new vessels produce an array of growth factors, including vascular endothelial growth factor (VEGF) and brain-derived neurotrophic factor, which promote angiogenesis and enhance survival and integration of neuroblasts into the surrounding cerebral tissue [6]. 

Despite the obvious need to re-establish blood flow to ischaemic brain tissue, reperfusion itself leads to further injury if blood reflow is not initiated within the first 1 to 2 h [9]. Spontaneous or thrombolytic reperfusion provides oxygen as a substrate for numerous enzymatic oxidation reactions turned on during ischaemia, several of which involve the excessive generation of toxic reactive oxygen species (ROS). However, in addition to their damaging effects in the hours after stroke studies our laboratory have now shown that ROS also play an important role in vascular remodeling [10,11,12,13] and ROS produced intracellularly serve as redox mediators of cell signaling in wound healing repair mechanisms such as angiogenesis [10,11,12,14]. Therefore, although ROS are involved in the progression of injury during the acute phase of stroke, they may also be vital participants in the later stages of brain repair, particularly in vascular signaling and cerebral angiogenesis.

The enzyme NADPH oxidase is the major source of ROS in the vascular system [15,16], inflammatory cells [16,17], cerebral blood vessels [18,19] and neurons [20,21]. The NADPH oxidase complex generates the highly reactive free radical, superoxide, via its Nox catalytic subunit [16], with Nox2 the active NADPH oxidase in inflammatory cells in both humans and animals. Two catalytic subunits separate to Nox2, are Nox1 and Nox4 and these are differentially expressed depending on tissue type, and play different roles in regulating ROS production [15,16]. We have previously characterized the temporal and cellular expression of NADPH oxidase subunits, Nox2 and Nox4, concurrently with increased superoxide generation and progression of damage in the acute phase of stroke and reperfusion in conscious rats (up to 7 days) [21]. However little is known about the role of NADPH oxidase in long term brain recovery. In this study we investigate the role of NADPH oxidase in brain angiogenesis after transient endothelin-1 (ET-1) induced ischemic stroke in rats. Our findings show increased temporal expression for both Nox2 and Nox4 NADPH oxidase mRNA in the long term recovery phase of stroke concurrently with increased angiogenesis within the damaged brain. Importantly we show evidence to suggest that new vessels within the ischaemic territory generate superoxide and are positive for increased Nox2 immunoreactivity suggesting a role for Nox2 mediated ROS in vascular remodeling after stroke, a process that may contribute to functional recovery detected in this animal model of stroke after 7 days.

## 2. Results and Discussion

### 2.1. Stroke Rating

A total of 52 rats were used for this study; 2 were excluded from analysis due to premature death outside the desired recovery period and 3 were excluded due to lack of observed behavioral responses during stroke induction. Only rats observed to circle clockwise and/or counter clockwise with clenched contralateral forepaw were included in this study to ensure that all experimental groups included in this study contained rats with a reliable degree of injury according to our previous methods [22]. Therefore a total of 47 rats were included for assessment in the current study.

### 2.2. Functional Assessments

**Figure 1:**
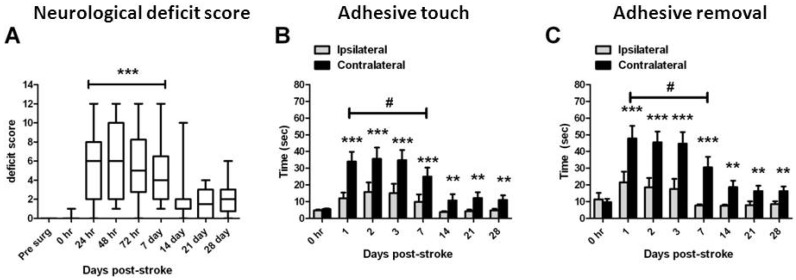
Long term neurological outcomes following ET-1 induced stroke. Combined neurological deficit scores (**A**) from all rats assessed in this study up to 28 days. Data presented as box plots include hinges extending from the 25th to 75th percentiles, the median line within the box and whiskers extending to the minimum and maximum values of the dataset. *** *p* < 0.001 compared with pre-stroke scores (non-parametric ANOVA). Latency to touch (**B**) and remove (**C**) a sticky label on the contralateral (stroke affected) forelimb compared with the ipsilateral forelimb from all rats assessed up to 28 days after stroke. Data presented as mean ± S.E.M. of time taken to touch each stimulus and then remove the stimulus. ** *p* < 0.01; *** *p* < 0.001 compared with the ipsilateral forelimb at the same time measurement (RM ANOVA); ^#^ *p* < 0.05 compared with the ipsilateral forepaw at 0 h (RM ANOVA). *n* = 35 assessed 1–3 days; *n* = 26 assessed day 7; *n* = 16 assessed day 14; *n* = 8 assessed days 21 and 28 for all behavior studies.

Neurological deficit scores were significantly increased from 24 h to 7 days post-stroke compared with pre-stroke scores (*p* < 0.05, non-parametric ANOVA; Figure 1A) but no significant deficits were detected after this time. Latency to touch (Figure 1B) and remove (Figure 1C) sticky labels was significantly increased in the contralateral forepaw at all times after stroke when compared with the ipsilateral forepaw at the same time (*p* < 0.001, two-way RM-ANOVA; Figure 1B,C). The time taken to remove a sticky label from the contralateral forepaw was significantly increased between 24 h and 7 days after stroke when compared to pre-stroke scores, but not at any time after 7 days (*p* < 0.05, two-way RM-ANOVA; Figure 1C).

### 2.3. Assessment of Damage

Morphological examination of infarcts in H&E stained sections from all recovery groups revealed damage in the parietal, insular, and frontal cortex as well as the dorsolateral striatum which extended through the corpus striatum as in previous studies [21,22]. Infarct volume appeared to reach maximum in the cortex by 3 days post-stroke and by 7 days in the striatum.

### 2.4. Angiogenesis after ET-1 Induced Stroke

**Figure 2:**
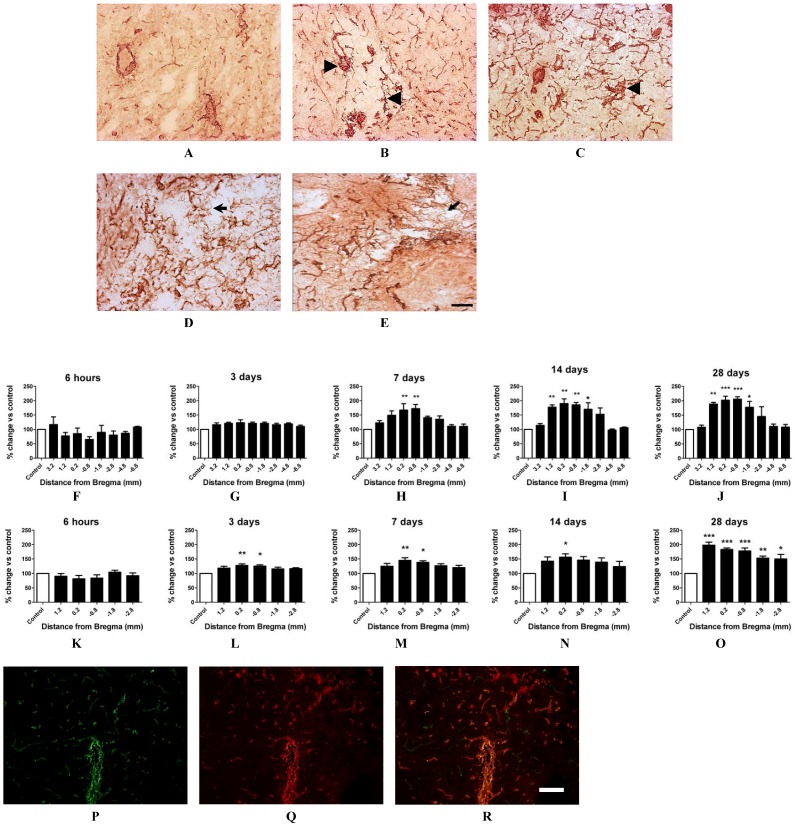
Angiogenesis in the ET-1 induced stroke affected brain. Immunohistochemical photomicrographs of vWF staining in the ipsilateral core cortex at 6 h, 3, 7, 14 and 28 days post-stroke (**A**–**E** respectively). Arrow heads indicate sprouting vessels and short arrows point to microvascular development by 14 and 28 days. Scale bar = 50 μM. Blood vessels were quantified *in situ* using vWF stained sections and point counted using Metamorph imaging software (**F–O**). A significant increase in the number of blood vessels was detected 7, 14 and 28 days after stroke in the ipsilateral core cortex (**H**–**J** respectively) and also at 3, 7, 14 and 28 days in the core ipsilateral striatum (**L**–**O** respectively) in comparison to respective contralateral mirror regions [6 h (*n* = 4); 3 days (*n* = 5); 7 days (*n* = 6); 14 days (*n* = 4); and 28 days recovery (n = 4)]. Data presented as mean ± S.E.M. * *p* < 0.05, ** *p* < 0.01, *** *p* < 0.001 compared with the contralateral side expressed as 100% control; one way ANOVA. Immunofluorescent photomicrographs of vWF (**P**) and lectin stain (**Q**). Merged images (**R**) show significant co-localisation of both vWF and lectin stain following lectin tail vein infusion indicative of vessel patency. Scale bar = 100 μm.

Using the vascular marker vWF *in situ*, blood vessels were visualised in the cortex and striatum across the core infarct (Figure 2) and within the surrounding border zone between 6 h and 28 days after stroke. Many enlarged vessels could be observed within the cortical infarct immediately after stroke (Figure 2A) with vascular sprouting apparent by 3 days and thin-walled micro-vessels detected by 14 and 28 days (Figure 2D,E). Blood vessel numbers in the contralateral cortex and contralateral striatum were point-counted as a measure of normal vascularization and expressed as 100% control. The number of blood vessels point-counted in each ipsilateral region was then expressed as a percentage of that in the corresponding contralateral region to determine the effects of stroke damage on angiogenesis. In the core cortex there appeared to be a reduction in vessel numbers close to the core infarct territory at 6 h post-stroke (1.2–0.8 mm Bregma, Figure 2F) but this did not prove significant. However a significant increase in blood vessel numbers was detected in the ipsilateral core cortex 7 days after stroke (~70% increase; *p* < 0.05, one way ANOVA, Figure 2H), with a marked increase in vessels detected both 14 and 28 days post-stroke now spanning throughout the infarct with peak vascularization observed by 28 days (~90%–110% increase respective; *p* < 0.05, one way ANOVA, Figure 2I,J). In the ipsilateral core striatum a significant increase in vessel numbers was detected as early as 3 days post-stroke (~28% increase; *p* < 0.05, one way ANOVA, Figure 2L) with a marked increase in vessel numbers detected by 28 days (~98% increase, *p* < 0.05, one way ANOVA; Figure 2L–O). Similar changes in cerebral vessels were also detected in the border zones of both the cortical and striatal infarcts, with peak vascularization in the border zone of the infarcted cortex detected between 14 and 28 days (data not shown). Tail vein injection of the vascular fluorescent marker lectin 10 min prior to tissue collection revealed that the majority of new vessels 28 days after stroke stained positive for both vWF and lectin, indicating patency of new vessels, with little evidence of vascular leakage as indicated by no obvious lectin staining outside of the vessels (Figure 2P–R). 

### 2.5. Changes in Brain Pathology Associated with Increased Angiogenesis

**Figure 3:**
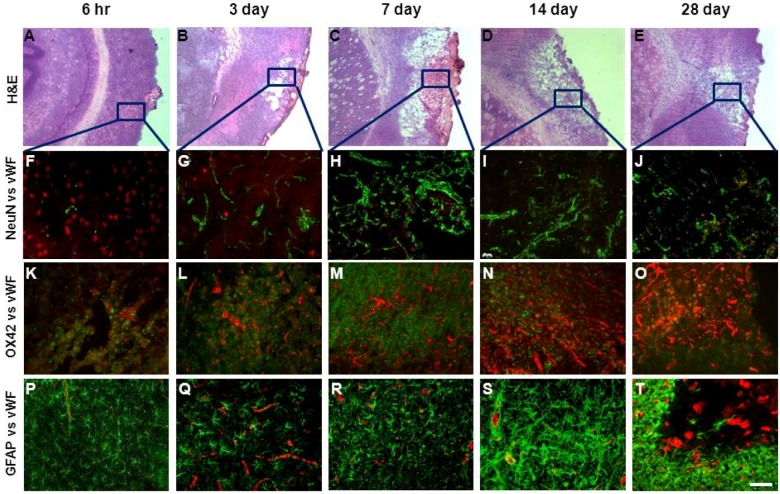
Angiogenesis and lesion pathology following ET-1 induced stroke. Hematoxylin and Eosin (H & E) stained images of the infarcted cortex between 6 h and 28 days post-stroke (**A**–**E**) were used to identify areas of interest for vWF, NeuN and OX42 histological analysis (as marked by frames). Merged immunofluorescent images of NeuN labeled neurons (red) and vWF labeled blood vessels (green) throughout the stroke affected cortex 6 h to 28 days after ET-1 induced stroke (**F**–**J**). Increased angiogenesis occurs in areas where there is greatest neuronal loss. Merged immunofluorescent images of vWF labelled blood vessels (red) with markers for microglia (OX42, green) (**K**–**O**); and astrocytes (GFAP, green) (**P**–**T**), 3, 7, 14 and 28 days post-stroke. Peak microglia activation occurs between 3 and 7 days whilst activated astrocytes are observed 3 days after stroke surrounding the damaged territory with intense staining detected by 14 days (**S**) that infiltrates into the core by 28 days (**T**). Scale bar = 100 μM.

Immunohistochemical co-localisation of neuronal marker NeuN and vWF revealed increased staining of new blood vessels in regions where there was greatest neuronal loss throughout the stroke affected brain. Increased blood vessels numbers in regions of the damaged cortex between 7 and 28 days post-stroke were associated with sustained neuronal loss over this time (Figure 3A–E). Analysis of inflammatory cell reactivity during the 28 day recovery period showed increased microglia/macrophage activation, evidenced by increased immunoreactivity to OX-42, across the infarcted cortex and striatum between 6 h and 7 days after stroke in regions also associated with increased vWF staining (Figure 3F–H). This effect appeared to subside by 14 and 28 days particularly in regions where peak angiogenesis was clearly evident (Figure 3I,J). Increased reactivity to the astrocytic marker GFAP occurred between 6 h and 3 days after stroke in the border zone to the infarct (Figure 3K,L), with intense staining detected by 14 days which extended into the core infarct region after this time. Although the temporal pattern for GFAP and vWF staining was similar, greatest levels of vWF staining occurred within the core infarct whereas increased GFAP immunoreactivity was mostly localised to the border zone (Figure 3N,O).

### 2.6. RT-PCR Detection of NADPH Oxidase Subunits and Angiogenic Factors

To investigate the temporal association between NADPH oxidase and angiogenesis, Nox isoforms and VEGF expression was determined by real-time PCR measures of mRNA for Nox2, Nox4, VEGF and related receptors. Serial samples were taken from the cortex and striatum to map changes throughout the brain following stroke. mRNA expression in the contralateral hemisphere 6 h after stroke was shown not to be significantly different to mRNA expression in the same brain regions of sham operated controls with 6 h and 3 days recovery (results not shown). Therefore comparisons over time were made in reference to contralateral level 1.2 from the 6 h stroke recovery group. Samples from the ipsilateral (stroke-affected) forebrain were compared with the contralateral (non-stroke affected) side, so that each rat acted as its own control. To allow for comparison of values across time points results from the cortex and striatum were expressed as a function of the appropriate mean ΔCt value associated with the contralateral side of the brain at 1.2 mm from Bregma in the 6 h recovery group 

#### 2.6.1. Change in mRNA in the Cortex

**Figure 4:**
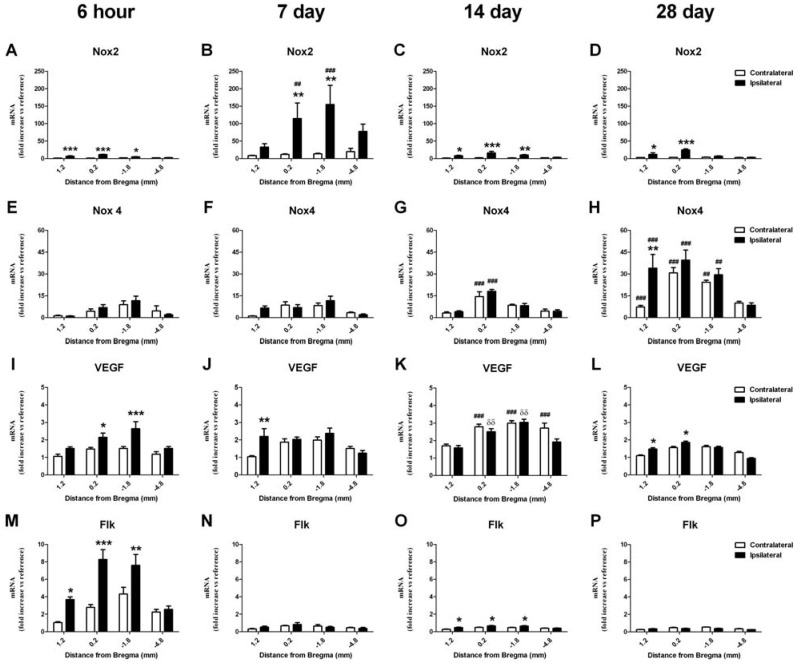
Real-time PCR detection of mRNA for NADPH oxidase subunits, angiogenic factor VEGF and its receptor, Flk, in the cortex 6 h to 28 days after ET-1 induced stroke. Nox2 mRNA expression is increased in the ipsilateral cortex as early as 6 h after stroke and remains elevated for up to 28 days in comparison to the contralateral hemisphere (**A**–**D**). Nox4 mRNA expression is also increased at 14 and 28 days post-stroke in both the ipsilateral and contralateral hemisphere in comparison to the 6 h group, with a greater increase detected in the ipsilateral hemisphere at 28 days (**E**–**H**). An increase in the angiogenic factor VEGF mRNA is detected in the ipsilateral cortex 6 hours after stroke and remains elevated for up to 28 days, with elevated levels also detected in the contralateral hemisphere at 14 days when compared to the 6 h group (**I**–**L**). An increase in VEGF receptor Flk mRNA after stroke is detected in the ipsilateral hemisphere but only at 6 h (**M**–**P**). Expression of mRNA was normalized to the respective GAPDH content for each sample (ΔCt), then expressed relative to the contralateral side of the brain at 1.2 mm from Bregma, 6 h post-stroke (ΔΔCt). Data are presented as mean SEM of 8 samples per region (*n* = 4 per group). * *p* < 0.05; ** *p* < 0.01; *** *p* < 0.001 *vs.* contralateral; ^#^ *p* < 0.05; ^##^ *p* < 0.01; ^###^ *p* < 0.001 *vs.* corresponding ipsilateral 6 h value; δδ *p* < 0.01 *vs.* corresponding contralateral 6 h value (ANOVA).

Nox2 mRNA expression was increased in the ipsilateral cortex in most brain regions at all times after stroke in comparison to the contralateral side (Figure 4A–D; *p* < 0.05, ANOVA). This increase was significantly greatest at 7 days post-stroke when compared to the contralateral control region in the 6 h recovery group (~150 fold increase; *p* < 0.05, two-way ANOVA). Nox4 mRNA expression was also significantly increased in the ipsilateral cortex 28 days after stroke in comparison to the contralateral side (Figure 4H; *p* < 0.05, ANOVA). However changes in Nox4 mRNA expression were also detected in the contralateral hemisphere at both 14 and 28 day recovery times in comparison to that originally detected in the contralateral control region in the 6 hour recovery group (*p* < 0.05, two-way ANOVA). Highest levels of Nox4 mRNA were detected 28 days post-stroke when compared to the contralateral control region in the 6 h recovery group (up to 40 fold increase; *p* < 0.05, two-way ANOVA). Increased levels of VEGF mRNA were detected in the ipsilateral cortex at all times after stroke (Figure 4I–L; *p* < 0.05, ANOVA) with changes also detected in the contralateral hemisphere at 14 days when compared to that originally detected in the contralateral control region in the 6 h recovery group (Figure 4K; *p* < 0.05, two way ANOVA). mRNA levels for VEGF receptors Flk and Flt were also investigated with no change in Flt detected at any time after stroke in the cortex. Increased levels of Flk mRNA were detected in the ipsilateral cortex 6 h after stroke but only at this time (Figure 4M; up to 7 fold increase; *p* < 0.05, ANOVA).

#### 2.6.2. Change in mRNA in the Striatum

**Figure 5:**
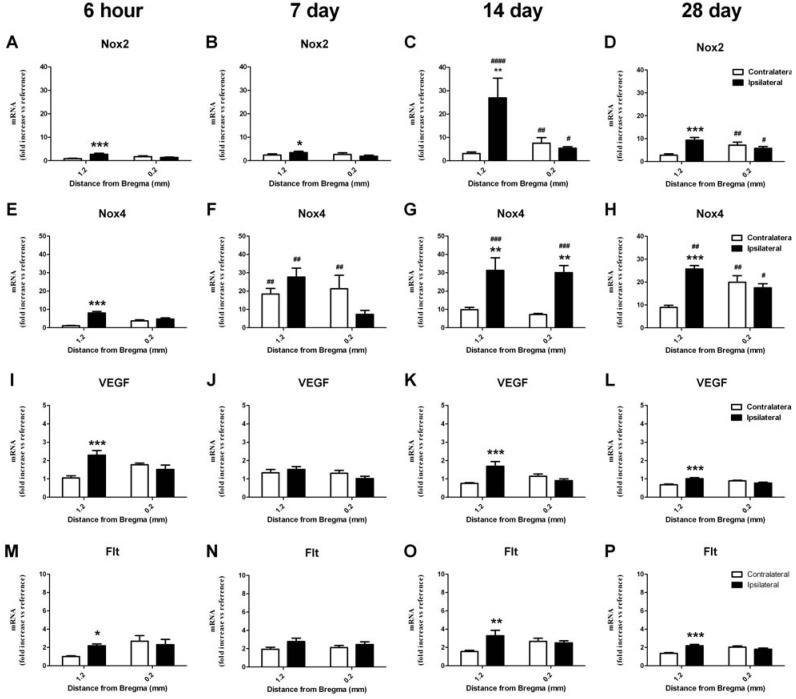
Real-time PCR detection of mRNA for NADPH oxidase subunits, angiogenic factor VEGF and its receptor, Flk, in the striatum 6 h to 28 days after ET-1 induced stroke. Nox2 mRNA expression is increased in the ipsilateral striatum as early as 6 h after stroke and remains elevated for up to 28 days in comparison to the contralateral hemisphere (**A**–**D**). Nox4 mRNA expression is also increased at 6 h in the ipsilateral striatum and remains elevated for up to 28 days post-stroke, with increases in both the ipsilateral and contralateral hemispheres detected at 7 and 28 days in comparison to the 6 h group (**E**–**H**). An increase in the angiogenic factor VEGF is detected in the ipsilateral striatum at 6 hand remains elevated for up to 28 days (**I**–**L**). An increase in VEGF receptor Flt mRNA after stroke is detected 6 h, 14 and 28 days after stroke in the ipsilateral hemisphere (**M**–**P**). Expression of mRNA was normalized to the respective GAPDH content for each sample (ΔCt), then expressed relative to the contralateral side of the brain at 1.2 mm from Bregma, 6 h post-stroke (ΔΔCt). Data are presented as mean SEM of 8 samples per region (*n* = 4 per group). * *p* < 0.05; ** *p* < 0.01; *** *p* < 0.001 *vs.* contralateral; ^#^ *p* < 0.05; ^##^ *p* < 0.01; ^###^ *p* < 0.001 *vs.* corresponding ipsilateral 6 h value; δ *p* < 0.05 *vs.* corresponding contralateral 6 h value (ANOVA).

Increased Nox2 mRNA expression was also detected in the ipsilateral striatum in the same brain region (bregma level 1.2) at all times investigated after stroke (Figure 5A–D; *p* < 0.05, ANOVA). This increase was significantly greatest in the 14 day recovery group when compared to the contralateral control region in the 6 h recovery group (~25 fold increase; *p* < 0.05, two-way ANOVA). Nox4 mRNA expression was also significantly increased in the ipsilateral striatum at all times investigated after stroke (Figure 5E–H; ~30 fold increase; *p* < 0.05, ANOVA) with changes in the contralateral hemisphere also detected at both 7 and 28 days in comparison to the contralateral control region in the 6 h recovery group (*p* < 0.05, two-way ANOVA). Increased levels of VEGF mRNA were detected in the ipsilateral striatum in the same brain region (bregma level 1.2) at 6 h, 14 and 28 day recovery times (Figure 5I–L; ~2 fold increase; *p* < 0.05, ANOVA). mRNA levels for VEGF receptors Flk and Flt were also investigated with no change in Flk detected at any time after stroke in the striatum. However increased levels of Flt mRNA were detected in the ipsilateral striatum 6 h, 14 and 28 days after stroke when compared to the contralateral control region in the 6 h recovery group (Figure 5 M–P; ~2 fold increase; *p* < 0.05, ANOVA).

### 2.7. Vascular Proliferation, Superoxide and Nox2 NADPH Oxidase

**Figure 6:**
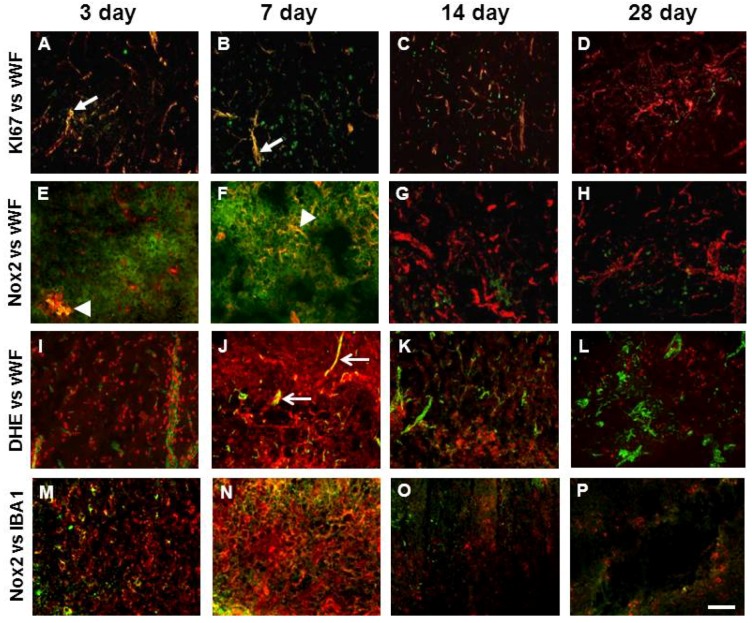
Nox2 immunohistochemistry in the stroke affected cortex is associated with proliferating blood vessels in addition to inflammatory cells. Merged immunofluorescent images of vWF labelled blood vessels (red) throughout the stroke affected cortex up to 28 days after ET-1 induced stroke co-localised with: the proliferation marker Ki67 (green) (**A**–**D**); a Nox2 antibody gp91Phox (green) (**E**–**H**); and the superoxide indicator DHE (**I**–**L**). Co-localisation (yellow) for Ki67 can be observed between 3 and 14 days after stroke (**A** and **B**, arrows) with little overlap observed by 28 days (**D**). Nox2 and vWF double labelling (yellow) can be seen 3 and 7 days after stroke (**E** and **F**, arrow heads) with little overlap detected beyond this time. DHE and vWF are also observed to co-localise at 3 and 7 days after stroke (**I** and **J**, open arrows). Merged immunofluorescent images of Nox2 (green) co-localisation with rabbit polyclonal IBA1 marker for activated microglia (red) (**M**–**P**). Scale bar = 100 μm.

**Figure 7:**
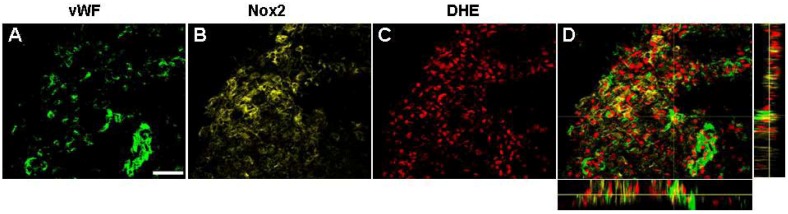
Newly formed blood vessels are positive for Nox2 and superoxide. Confocal microscopy of triple labelled immunofluorescence for vWF (green) (**A**), Nox2 (yellow) (**B**), and DHE (red) (**C**), in the damaged cortex shows new blood vessels are positive for both Nox2 and superoxide 7 days post stroke (Orthogonal view **D**). Images were captured as collapsed reconstructions of optical sections every 0.2 μm on the *z*-axis. Scale bar = 50 μm

Immunofluorescence to the proliferation maker Ki-67 was detected in the ipsilateral cortex and striatum between 3 and 14 days post-stroke where it co-localised occasionally with vWF labelled blood vessels indicative of vascular proliferation (Figure 6A–C), an effect that was most prominent at 3 and 7 days and no longer detected by 28 days (Figure 6D). Nox2 immunoreactivity was examined throughout the brain where increased angiogenesis was detected at all times investigated after stroke. Increased Nox2 immunoreactivity was clearly visualised in the ipsilateral core cortex up to 14 days after stroke and was mostly associated with cell membranes of phagocytic like cells (Figure 6). Although Nox2 immunohistochemistry was mostly associated with IBA1 positive cells, Nox2 was all observed to be double labelled with vWF between 3 and 7 days after stroke and associated with vascular sprouting and small microvessels (Figure 6E,F). Newly generated blood vessels in the damaged cortex were also positive for the superoxide indicator DHE at both 3 and 7 days post-stroke (Figure 6I,J). Triple labelling for Nox2, DHE and vWF was viewed using a confocal microscope and revealed co-localisation for all three markers between 3 and 7 days post-stroke (Figure 7D).

### 2.8. Discussion

In the present study we show that angiogenesis commences between 3 and 7 days after endothelin-1 induced stroke in conscious rats, with new vascular networks largely detected in regions where there was severe and sustained neuronal loss. Vascular sprouting in the damaged cortex between 3 and 7 days was associated with superoxide generation as well as Nox2 labeling, and changes in cortical Nox2 mRNA were most significant at these recovery times. Data also revealed changes in Nox4 mRNA up-regulation in the ipsilateral cortex and striatum between 7 and 28 days after stroke concurrently with the appearance of new vascular networks and changes in vascular growth factors. These data collectively indicate a potential role for NADPH oxidase in the development of new vasculature within the damaged brain in the recovery phase after stroke, and highlight specifically a role for Nox2 in brain repair. 

#### 2.8.1. Angiogenesis following ET-1 Induced Stroke

There is little evidence to suggest that significant improvements in neurological function detected by 14 days after stroke in the present study is attributed to neuronal replacement within the damaged brain and recovery is more likely due to brain plasticity, rewiring or recovery of depressed neuronal pathways reported by others [23]. However the potential for brain regeneration relies heavily on the surrounding microenvironment and if neuronal replacement was to be achieved it would require supporting vasculature. In rats, stroke increases the percentage of proliferating stem cells within the subventricular zone that contribute to neurogenesis as soon as 2 days after stroke, an effect that has been reported to peak by 7 days, and returns to normal by 14 days [24]. In the present study we show that, although angiogenic factors are up-regulated by 7 days, new vascular networks are not evident until 14 days. So, although vascular remodeling commences early [6], the developing vasculature is not mature enough in the early phase of neurogenesis to support neuronal replacement. Indeed it has recently been reported that neuroblast survival is dependent on mature vascular network formation [25] and in the present study we show that new vascular networks capable of perfusion are not evident until 14 days after stroke when the endogenous neurogenic response is reported to subside [24].

Alternatively Yu and colleagues, 2007 [26] report that new vascularization following stroke in rats is only transient and that after 30 days vessel hyper density regresses significantly and these authors suggest that the role of angiogenesis is for the removal of damaged tissue and does not support tissue replacement [26]. In the present study we show that neurons are lost by 7 days with little evidence of neuronal debris by 14 days. Hence it is unlikely that the degree of angiogenesis detected in this study between 14 and 28 days is purely related to removal of debris and we suggest that angiogenesis may be important for other repair process. If these repair processes are not achieved then vessel density may later regress. Mechanisms associated with angiogenesis need to be explored so that this process may be stimulated to occur sooner in order to support neuronal replacement [25]. Indeed recent studies show that therapeutic angiogenesis enhances neurogenesis and functional recovery to support brain remodeling [4,25,27].

#### 2.8.2. NADPH Oxidase and Angiogenesis

In addition to their damaging effects in the hours after stroke, we have shown that NADPH oxidase derived ROS in other models of tissue engineering play an important role in vascular remodeling [11,12,15,27] and ROS produced intracellularly serve as redox mediators of cell signaling in wound healing repair mechanisms such as angiogenesis [10,11,13,28,29]. Nox-2 derived ROS have been shown to be key mediators of compensatory neovascularisation in response to hind limb ischemia where an increase in ROS generation was detected initially from infiltrated inflammatory cells and subsequently from endothelial cells, with Nox2 as the major source of ROS from these two cell types [30]. Additionally, absence of a functional Nox2 in knockout mice reduces VEGF production by 50% following hind limb ischemia, indicating a role for Nox2 in regulating signaling events for neovascularisation downstream [30]. Here we report for the first time evidence to show that regenerating blood vessels in the rat brain after stroke are positive for Nox2 and superoxide suggesting that in addition to meditating injury, Nox2 may also play a role in brain repair. This is important to consider when targeting therapies that inhibit Nox2 for the prevention of injury, which may also inadvertently inhibit brain repair. 

The spatiotemporal expression of Nox2 and Nox4 in the acute phase of stroke has been well published previously with a major focus on their contribution to brain injury [20,21,31]. We have previously characterized in detail the up-regulation of both Nox2 and Nox4 in the acute phase of stroke with reperfusion in conscious rats (up to 7 days recovery) [21]. This occurs concurrently with increased superoxide generation in neurons (6–24 h) and later within Nox2 labelled activated microglia/macrophages (24 h to 7 days). Furthermore we have also shown that NADPH oxidase-derived ROS are increased in damaged blood vessels within the ischemic core and penumbra 3 days following stroke and reperfusion [19] suggesting that the cerebral vasculature may also be an important source of ROS in rats after stroke. However until recently there have been few studies that have investigated a potential role for NADPH oxidase in brain recovery. Here we show for the first time that in addition to inflammatory cells, Nox2 is co-localised to blood vessel clusters generating superoxide in the damaged cortex between 3 and 7 days after stroke prior to the formation of new vascular networks that are evident by 14 days. We therefore suggest that vascular Nox2 generated ROS may play a role in vessel sprouting and elongation in the initial phase of angiogenesis, and once new vascular networks are established, Nox2 signaling subsides. A lack of Nox2 immunoreactivity within vessels, together with reduced Nox2 mRNA expression beyond 14 days when vascular sprouting subsides, further supports this. This is not the first instance we have reported a role for Nox2 in vascular sprouting. Using a chamber model of arterial venous loop re-construction, we have shown a high level of Nox2 expressed in endothelial cells of newly developed blood vessels sprouting from the loop and local administration of Nox inhibitors apocynin or gp91ds-tat peptide to the chamber suppressed angiogenesis [11]. It will be important to focus future studies on investigating the effects of long term treatments with specific Nox2 inhibitors or Nox2 deficient mice on angiogenesis after stroke to confirm the current findings.

Increased Nox2 expression was also clearly associated with inflammatory cells after stroke. It is increasingly evident that ROS generation from inflammatory cells contributes to brain injury, however it is unlikely that Nox2 generated ROS beyond 7 days contributes significantly to cellular damage or death as neurons are lost within the ischaemic core by this time. ROS produced at this time could however serve as redox mediators of cell signaling in brain repair mechanisms. Indeed inflammatory cells play a vital role in the repair process through release of a variety of trophic factors and signaling molecules which may contribute to angiogenesis. For this reason blocking ROS indiscriminately in the recovery phase of stroke may be detrimental to brain repair mechanisms. Given the recent focus on developing antioxidants for prevention of stroke injury, it is important to understand the role of ROS generating enzymes beyond the initial stroke insult in order to maximize functional recovery beyond the acute phase of stroke.

In addition to early changes in Nox2, Nox4 is also up-regulated after stroke concurrently with increased superoxide generation [20,21,32]. Nox4 has recently been reported as the most important NADPH oxidase homologue in a mouse model of focal ischaemic stroke, with Nox4^−/−^ mice exhibiting less damage and less ROS generation with 24 hours recovery [32]. These effects were mostly associated with both neuronal and vascular Nox4 expression [32]. Compared with peripheral vessels, expression of Nox4 and ROS production is 10-100 times higher in cerebral vessels [18] and Nox4 is localised initially to cortical neurons and later to newly formed capillaries 4 weeks after permanent stroke in mice [20]. We now show for the first time that Nox4 is also up-regulated in the damaged rat brain in the weeks after stroke and reperfusion, concurrently with Nox2 and the most important pro-angiogenic factor, VEGF. Following stroke, VEGF is expressed in neurons for days and for up to 2 weeks in astrocytes [33]. Interestingly changes in both Nox4 and VEGF were also detected in the contralateral hemisphere and are similar to that which we’ve seen before where NADPH oxidase activity in cerebral blood vessels contralateral to the damaged brain was increased in comparison to vessels from sham rats [19]. These changes in the contralateral brain however do not correlate with changes in vascular numbers with no evidence of angiogenesis such as vessel sprouting in the contralateral hemisphere. Changes in the contralateral hemisphere may however represent an attempt at endogenous neuroprotection and Nox 4 has recently been reported to protect the vasculature during ischemia and inflammatory stress [34]. Additionally changes in receptors for VEGF such as Flk were only up-regulated in the damaged hemisphere after stroke and this could account for why angiogenesis was detected in the ipsilateral hemisphere alone. To support this, recent studies have shown that Flk-1 is associated with endothelial cells and angiogenesis following brain injury [35]. Additionally Flt-1, shown to be associated with astrocytes, was, in this study found to be increased in the ipsilateral striatum where angiogenesis appeared to be delayed. Interestingly, the striatum was an area where intense scar formation appeared by 14–28 days and recent studies suggest a role for flt-1 signaling in scar formation after brain injury.

What role Nox4 might have in the damaged brain during recovery is still yet to be determined. Craige and colleagues 2011 [31] recently showed that neovascularisation following hind limb ischemia was accelerated in mice overexpressing Nox4, thus further supporting a role for NADPH oxidase mediated ROS in vascular recovery. However in the present study increased Nox4 expression was only detected after new vascular networks had formed and similarly others have reported increased Nox4 in new capillaries in mice after 4 weeks [20]. Additionally we have previously shown using human endothelial cells that blocking Nox4 prevents endothelial tube formation suggesting at role for Nox4 in vessel maturation [10]. If this is true for Nox4 in cerebral angiogenesis, treatments that stimulate Nox4 expression/activity in the weeks after stroke may accelerate vascular recovery and reduce blood-brain barrier permeability that is often associated with the early stage angiogenesis [36]. In our hands the lack of a reliable Nox4 antibody to accurately identify Nox4 at a cellular level makes it difficult to determine where indeed increased expression of Nox4 is localised. Although in this study the temporal regulation of Nox4 correlated with maturation of new vascular networks, it is still difficult to determine if Nox4 is playing a role in mediating angiogenesis or whether there is more Nox4 present because there are increased vessel numbers. 

## 3. Experimental Section

### 3.1. Ethics Statement

All experiments described were performed in accordance with the Prevention of Cruelty to Animals Act 1986 under the guidelines of the National Health and Medical Research Council Code of Practice for the Care and Use of Animals for Experimental Purposes in Australia. The protocol was approved by the St Vincent’s Hospital Animal ethics committee (AEC 009/09). All surgery was performed under general anesthesia, and all efforts were made to minimize suffering which included access to paracetamol (2 mg/kg in drinking water) for 24 h prior to and following surgery, as well as extensive monitoring of each rat throughout the duration of the study. 

### 3.2. Endothelin-1 Stroke in Conscious Rats

Male hooded Wistar rats, aged 10–12 weeks (300–340 g) were anesthetized with Ketamine/Xylazine (75 mg/kg Ketamine/10 mg/kg Xylazine i.p.) and maintained throughout surgery by inhalation isoflurane (95% oxygen and 2% isoflurane). A 23-gauge stainless-steel guide cannula was stereotaxically implanted into the piriform cortex 2 mm dorsal to the right middle cerebral artery (0.2 mm anterior, −5.2 mm lateral, and −5.9 mm ventral) as described previously [20,21] for delivery of ET-1 to induce focal ischemia in conscious rats at a later time. 

Five days post-surgery constriction of the right middle cerebral artery was induced in conscious rats by perivascular administration of ET-1 (American Peptide Company; 20 pmol in 2 µL saline over 10 min) [21,22]. During stroke induction, clockwise and/or counter clockwise circling with clenching and dragging of the contralateral forepaw were observed, validating correct placement of the cannula [21,22]. All rats included in this study (*n* = 47) showed significant signs of stroke based on these behavioural changes observed within 2–60 min of the commencement of the ET-1 injection as described previously [21]. For real time PCR studies control rats (sham; *n* = 4 at 6 h and *n* = 4 at 3 day recovery) underwent cannula implantation and saline infusion instead of endothelin-1.

### 3.3. Assessment of Functional Outcome

Behavioural tests were conducted in all groups in order to assess the effect of stroke damage on functional outcomes as in our previous studies [18,21]. All rats were coded so that the investigator was blinded to treatment condition. Assessments were conducted on all rats, prior to any procedures (pre-surgery), immediately prior to ET-1-induced vasoconstriction (pre-ischemia), and 24, 48, and 72 h, 7, 14, 21 and 28 days after stroke where appropriate. The behaviour of each rat was compared with pre-stroke scores, so each rat acted as its own control. Neurological abnormalities were evaluated with the use of a neurological deficit score and asymmetry was evaluated by using the sticky label test as previously described [21]. 

### 3.4. Confirmation of Ischemic Damage

Rats were decapitated 6 h (*n* = 4), 3 (*n* = 5), 7 (*n* = 6), 14 (*n* = 4) or 28 days (*n* = 4) after ischemia and brains were removed and frozen in liquid nitrogen and stored at −80 °C. Coronal cryostat sections (16 μm) were cut at eight predetermined coronal planes throughout the brain from −3.2 to 6.8 mm relative to Bregma. Sections were stained with Hematoxylin and Eosin (H&E) for morphological examination of infarcts in all groups. 

### 3.5. Blood Vessel Detection

For detection of blood vessels adjacent sections to those used to confirm damage were fixed with methanol at −20 °C for 15 min and washed in PBS (0.1 M) containing 0.1% v/v tween 20 detergent (PBT, 2 × 5 min) followed by a 30 min pre-block in 10% normal goat serum in PBT. Slides were then incubated for 1 h with rabbit polycolonal anti-human von Willebrand factor (vWF) (1:200; Chemicon, Temecula, CA, USA) prior to wash in PBS (0.1 M; 2 × 5 min) and 30 min incubation with biotinylated anti-rabbit IgG (1/200; Vector Labs, Burlingame, CA, USA) and analysed using an ABC detection kit (Vector). The resultant colour reaction was visualised with a Zeiss Axioskop2 microscope using brightfield settings. Analogous experiments were also conducted with goat anti-rabbit IgG control serum and omission of the primary antibody from the protocol.

### 3.6. Blood Vessel Quantification

Blood vessel quantification was assessed blinded to recovery group in vWF-labelled sections across eight predetermined coronal planes (−3.2 to 6.8 mm relative to Bregma). At least three sections per region were used for assessment. vWF-stained sections were viewed using an Olympus (Tokyo, Japan) microscope under 20× magnification. Using H&E stained sections as a reference to confirm damage; four regions of interest were identified in each rat for assessment: the damaged cortex, damaged striatum, as well as the border zone surrounding the damage for both the striatum and cortex. Each region was then compared with the appropriate corresponding mirror region in the contralateral hemisphere using the exact same sample area. Using digital video imaging (TK C 1480E; JVC, Wayne, NJ, USA), an automated systematic random sampling point-counting system was applied using the Computer Assisted Stereological Toolbox (CAST System; Olympus, Mount Waverley, Australia) [37]. Sampling commenced randomly within each defined field and the number of points that overlay blood vessels was scored, and then divided by the total number of points counted in each field. The number of blood vessel detected in the ipsilateral region of interested was then compared to the corresponding contralateral side, which was expressed as 100% control.

### 3.7. Fluorescent Immunohistochemistry

In order to characterize cell specific changes during the recovery phase of stroke in regions where angiogenesis was detected dual fluorescent immunohistochemical techniques were employed. Frozen slide mounted sections were fixed in 4% PFA for 10 min. All sections were subject to a pre-block for 1 h in DAKO universal Blocking solution, and then washed in phosphate buffered saline (2 × 5 min PBS, 0.1 M, pH 7.4). Sections were then incubated overnight at 4 °C with rabbit anti-human vWF (1:200) in a mixture of PBS (0.1 M, pH 7.4) containing 2% Normal Goat Serum (NGS), 2% Normal Donkey Serum (NDS) and 0.3% Triton-X, together with either: neuronal mouse monoclonal anti-NeuN (1:400; Chemicon, Temecula, CA, USA) for detecting mature neurons; mouse monoclonal anti-Ki67 (1:500; Novocastra, North Ryde, Australia ) a marker of cellular proliferation; mouse monoclonal antibody MRC OX-42 (1:100; Serotec, Raleigh, NC, USA) a marker of activated microglia/macrophages; or mouse monoclonal anti-GFAP (1:400; Millipore, Kilsyth, Australia) a marker of astrocytes. Tissue sections were then washed in PBS (0.1 M, pH 7.4, 3 × 10 min) and transferred for incubation with an appropriate secondary antibody: AlexaFluor 568 goat anti-mouse IgG (1:500) and AlexaFluor 488 donkey anti-rabbit IgG (1:500) in a mixture of PBS (0.1 M, pH 7.4) containing 2% NGS, 2% NDS and 0.3% triton-X for 2.5 h at room temperature. Following incubation with the secondary antibodies, tissue sections were washed in PBS (0.1 M, pH 7.4; 2 × 5 min) and then cover-slipped using fluorescent mounting medium (Dako Cytomation, Carpinteria, CA, USA). Analogous experiments were also conducted with appropriate IgG control serum in place of primary antibodies. Resulting sections were examined with a fluorescence microscope equipped with a 578–603 nm filter set for detection of red fluorescence and a 495–519 nm filter set for the detection of green fluorescence (ZeissAxioskop2, North Ryde, Australia).

### 3.8. Nox Immunohistochemistry

Immunoreactivity to a Nox2 primary antibody was assessed using both light field and fluorescent microscopy. For light field microscopy sections were first fixed in acetone (50% 2 min, 100% 2 min, 50% 2 min) followed by incubation with mouse monoclonal anti-gp91Phox (1:500; BD Biosciences, San Jose, CA, USA) and analyzed using a standard HRP based immunoassay detection system with 3-Amino-9-ethylcarbazole (AEC) substrate (Chemicon). Analogous experiments were also conducted with mouse IgG control serum in place of the primary antibody. The color reactant was visualized with a Zeiss Axioskop2 microscope using brightfield settings. 

For dual fluorescence studies, sections were first fixed in 4% PFA for 10 min and then subjected to a pre-block for 1 h in DAKO universal Blocking solution and then washed in phosphate buffered saline (2 × 5 min PBS, 0.1 M, pH 7.4). Sections were incubated with anti-gp91phox together with either anti-human vWF (1:200) or mouse monoclonal antibody MRC OX-42 (1:100) in a mixture of PBS (0.1 M, pH 7.4) containing 2% NGS, 2% NDS and 0.3% Triton-X overnight at 4 °C. Tissue sections were then washed in PBS (0.1 M, pH 7.4, 3 × 10 min) and transferred for incubation with secondary antibodies AlexaFluor 568 goat anti-mouse IgG (1:500) and AlexaFluor 488 donkey anti-rabbit IgG (1:500) and processed using the same protocol as described above for fluorescence immunohistochemisty.

**Figure 8:**
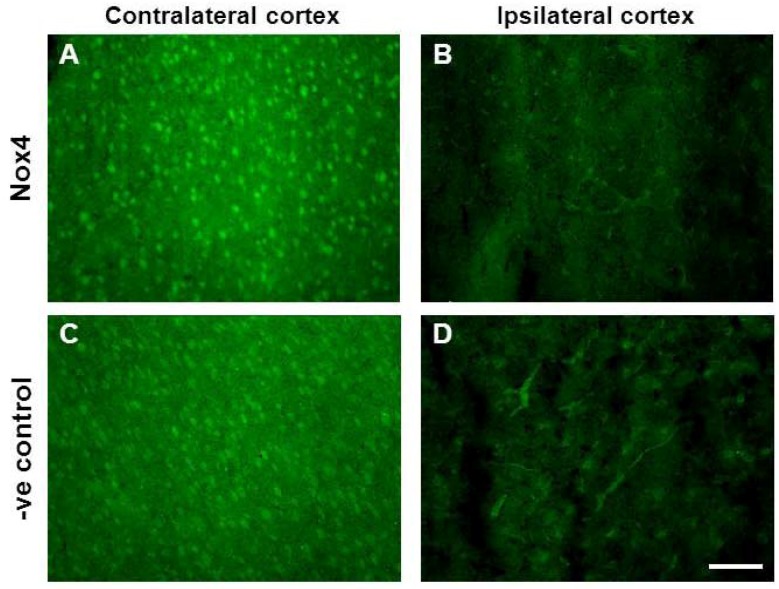
Nox4 immunohistochemistry in the ipsilateral cortex 28 days after stroke. Immunofluorescent images of rabbit monoclonal Nox4 antibody (Epitomics) in the contralateral (**A**) and ipsilateral (**B**) (Stroke affected) cortex 28 days post-stroke. Adjacent immunofluorescent images of control rabbit IgG serum in the contralateral (**C**) and ipsilateral (**D**) cortex reveal a high degree of non-specific immunoreactivity to blood vessels and neurons alike. Scale bar = 100 μm.

Immunoreactivity to a Nox4 primary antibody was also attempted. Adjacent sections were fixed in 4% PFA for 10 min prior to pre-block for 1 h in DAKO universal Blocking solution. Tissue sections were then washed in phosphate buffered saline (2 × 5 min PBS, 0.1 M, pH 7.4) and transferred for overnight incubation at 4 °C with rabbit monoclonal Nox4 antibody (1:200; Epitomics, Burlingame, CA, USA) in a mixture of PBS (0.1 M, pH 7.4) containing 2% NGS and 0.3% Triton-X. Sections were again washed (2 × 5 min PBS) and transferred for incubation with the secondary antibody Alexa 488 goat anti-rabbit (1:500) containing 2% NGS and 0.3% triton-X for 2.5 h at room temperature. Tissue sections were then washed in PBS (0.1 M, pH 7.4; 2 × 5min) and cover-slipped using fluorescent mounting medium (Dako Cytomation, Carpinteria, CA, USA). Analogous experiments were also conducted with rabbit IgG control serum in place of the primary antibody. Resulting sections were examined with a fluorescence microscope as described above. Unfortunately all attempts to localise Nox4 immunoreactivity using a specific Nox4 antibody were unsuccessful with sections incubated in the presence of the primary antibody revealing a similar pattern of distribution as sections exposed to IgG control serum in the absence of the primary antibody (Figure 8).

### 3.9. In Situ Detection of Superoxide Using Dihydroethidium Fluorescence

The oxidative fluorescent indicator dihydroethidium (DHE; Molecular Probes, OR) was used to detect superoxide *in situ* as previously described [20]. Briefly, slides adjacent to those used in fluorescent immunohistochemical studies were incubated with DHE (2 µM in 0.1% DMSO, 0.1 M PBS, pH 7.4) in a humidified chamber and protected from light for 30 min at 37 °C. Adjacent sections were also first incubated with vWF and processed as above for fluorescent immunohistochemistry prior to incubation with DHE. The resulting sections were examined with a fluorescence microscope (Zeiss Axioskop2) additionally equipped with a 590 nm long-pass filter set.

### 3.10. RT-PCR Detection of NADPH Oxidase Subunits and Angiogenic Factors

In separate groups of animals used for the above histological studies, rats were decapitated 6 h (*n* = 4), 7 day (*n* = 4), 14 days (*n* = 4) and 28 days (*n* = 4) after ischemia, their forebrains removed and cut into 4 × 2 mm coronal sections, using a brain matrix, corresponding to distances 1.2, 0.2, −1.8, and −4.8 mm relative to Bregma. For sham control rats forebrains were harvested 6 h following saline infusion (*n* = 4). Each segment was divided into contralateral and ipsilateral hemispheres and frozen over liquid nitrogen prior to further dissection of cortex and striatum over dry ice. The time between removal of the brain and freezing was approximately 2 min. These segments were then homogenized in 500 μL of TRI Reagent (Ambion, Austin, TX, USA) using an ultrasonic liquid processor (Microson™ XL 2000; Misonix, New York, NY, USA), and total RNA was extracted according to the manufacturer’s instructions. Reverse transcription was carried out with Taqman Reverse Transcription Reagents (Applied Biosystems, Foster City, CA, USA) using RNA (100 ng) in a final reaction volume of 10 μL. Real-time PCR and the ΔΔCt method were used as previously described to examine mRNA expression of the flavin- containing NADPH oxidase subunits Nox2 and Nox4 relative to a “reference” sample [20]. 5′-FAM-labeled Assays on Demand (AoDs) for target genes were purchased from Applied Biosystems. GAPDH was used as the internal standard for each reaction and was detected with a commercially available VIC labelled assay (Applied Biosystems). Threshold cycle (Ct) values for each test gene were normalized to the Ct values for the 18 s control from the same cDNA preparations. ΔΔCt was calculated using the contralateral side of the brain at 1.2 mm from Bregma, 6 h post-stroke, to allow for comparison between time points.

### 3.11. Statistical Analysis

Neurological outcome data was analysed by non-parametric Kruskal-Wallis test. Hemineglect data were analyzed by two-way RM ANOVA with two-factor repetition (side 3 h after stroke) to compare latencies in the ipsilateral and contralateral forepaws over time. Infarct volume, blood vessel quantification, and PCR data were analysed by two-way ANOVA for comparison between ipsilateral and contralateral sides, or for comparison between recovery times in PCR studies. Individual comparisons were made using Tukey’s test for all analyses where ANOVA yielded a significant result. We have previously shown that *n* = 4 are required for 80% power where a 2 fold change in mRNA expression is considered significant with a SD of approximately 1 for each group [21]. All data were analysed using GraphPad Prism Version 5.04, 1992–2010, GraphPad Software Inc., La Jolla, CA, USA.

## 4. Conclusions

Collectively these data challenge the view that brain ROS and specific NADPH oxidases are solely deleterious after stroke and support a role for Nox2 in brain repair mechanisms, particularly vascular signaling, sprouting and angiogenesis in the weeks after the damage has occurred. Many reports have shown that angiogenesis is a common occurrence in regions of the brain that border the infarct after stroke, but here we show that much angiogenesis occurs within the ischemic infarct with the potential to support brain repair and remodeling through delivery of oxygen and nutrients. Therefore promoting angiogenesis early after stroke may be a necessary factor to consider in conjunction with neurorestorative therapies that support or promote endogenous neurogenesis. Understanding the role of specific NADPH oxidases in angiogenesis following ischaemic stroke will enable development of treatment strategies that aim to protect the brain in the initial phase of stroke injury, but also promote angiogenesis through specific Nox simulation in the weeks after stroke to support endogenous repair and accelerate improvement in functional outcomes.
